# Human T-bet^+^ B cell development is associated with BTK activity and suppressed by evobrutinib

**DOI:** 10.1172/jci.insight.160909

**Published:** 2022-08-22

**Authors:** Liza Rijvers, Jamie van Langelaar, Laurens Bogers, Marie-José Melief, Steven C. Koetzier, Katelijn M. Blok, Annet F. Wierenga-Wolf, Helga E. de Vries, Jasper Rip, Odilia B.J. Corneth, Rudi W. Hendriks, Roland Grenningloh, Ursula Boschert, Joost Smolders, Marvin M. van Luijn

**Affiliations:** 1 Department of Immunology and; 2 Department of Neurology, MS Center ErasMS, Erasmus MC, University Medical Center Rotterdam, Rotterdam, Netherlands.; 3 Department of Molecular Cell Biology and Immunology, Amsterdam UMC, MS Center Amsterdam, Amsterdam Neuroscience, Amsterdam, Netherlands.; 4 Department of Pulmonary Medicine, Erasmus MC, University Medical Center Rotterdam, Rotterdam, Netherlands.; 5 EMD Serono, Billerica, Massachusetts, USA.; 6 Ares Trading SA, Eysins, Switzerland (an affiliate of Merck KGaA, Darmstadt, Germany).

**Keywords:** Autoimmunity, Immunology, Adaptive immunity, Antigen-presenting cells, Chemokines

## Abstract

Recent clinical trials have shown promising results for the next-generation Bruton’s tyrosine kinase (BTK) inhibitor evobrutinib in the treatment of multiple sclerosis (MS). BTK has a central role in signaling pathways that govern the development of B cells. Whether and how BTK activity shapes B cells as key drivers of MS is currently unclear. Compared with levels of BTK protein, we found higher levels of phospho-BTK in ex vivo blood memory B cells from patients with relapsing-remitting MS and secondary progressive MS compared with controls. In these MS groups, BTK activity was induced to a lesser extent after anti-IgM stimulation. BTK positively correlated with CXCR3 expression, both of which were increased in blood B cells from clinical responders to natalizumab (anti–VLA-4 antibody) treatment. Under in vitro T follicular helper–like conditions*,* BTK phosphorylation was enhanced by T-bet–inducing stimuli, IFN-γ and CpG-ODN, while the expression of T-bet and T-bet–associated molecules CXCR3, CD21, and CD11c was affected by evobrutinib. Furthermore, evobrutinib interfered with in vitro class switching, as well as memory recall responses, and disturbed CXCL10-mediated migration of CXCR3^+^ switched B cells through human brain endothelial monolayers. These findings demonstrate a functional link between BTK activity and disease-relevant B cells and offer valuable insights into how next-generation BTK inhibitors could modulate the clinical course of patients with MS.

## Graphical abstract

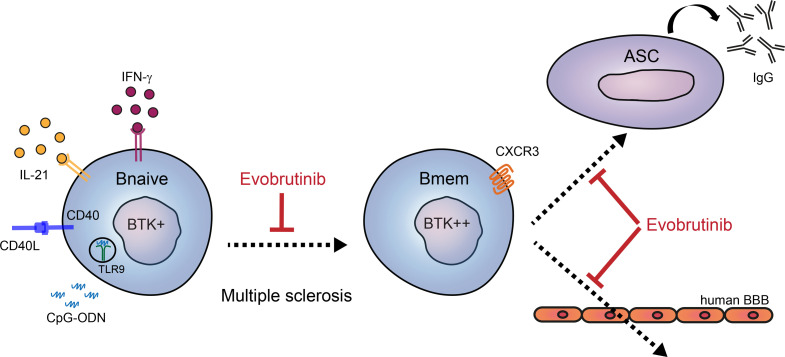

## Introduction

B cells play a detrimental role in the pathophysiology of autoimmune and chronic inflammatory diseases, including multiple sclerosis (MS) (1). In MS, this is exemplified by the presence of unique oligoclonal IgG fractions in the cerebrospinal fluid and the high efficacy of anti-CD20 monoclonal antibody B cell–depleting therapies (2). Because B cells are required for protective immune responses, their long-term depletion by such therapies puts patients at risk of infectious complications. More functionally restricted therapeutic strategies are needed to target B cell subsets that contribute to diseases such as MS. To accomplish this, it is critical to understand how extrinsic and intrinsic signals guide the development of these B cells in humans.

It is probable that, after their escape from peripheral tolerance checkpoints (3), naive B cells from patients with MS differentiate into memory populations capable of stimulating proinflammatory CD4^+^ T cells to enter the CNS (4). These CNS-homing CD4^+^ T cells respond to B cell–tropic EBV (5) and produce high levels of IFN-γ but not IL-17 (4, 6, 7). Concomitantly, the peripheral interaction between B and T cells likely contributes to the enhanced CNS recruitment of B cells expressing CXCR3 in MS (8).

CXCR3 expression on B cells is triggered by T-box transcription factor, T-bet (9). In mice, IFN-γ production by CD4^+^ T cells is essential for the induction of T-bet in autoreactive B cells within germinal centers (10). IFN-γ synergizes with pathogen-associated TLR9 ligands to induce T-bet expression and autoimmune-like responses (11). B cells from patients with MS are highly sensitive to both triggers (12), resulting in enhanced class switching (8). Moreover, CXCR3^+^ memory B cells are triggered by EBV (13) and show pronounced CNS-infiltrating ability and recall responses in vitro (8, 13, 14). This points to a tendency of these B cells to further mature into antibody-secreting cells (ASCs) after entering the CNS, a process associated with less favorable outcomes in MS (15).

Bruton’s tyrosine kinase (BTK) inhibitors, which are currently first-line treatments for chronic lymphocytic leukemia (16), represent an emerging class of compounds for the treatment of autoimmune diseases (17, 18). BTK is known for its role as a key signaling molecule downstream of the B cell receptor (BCR) that regulates cell proliferation and survival. Additionally, it is involved in other pathways, including TLR9 signaling to stimulate B cell–driven autoimmunity (11, 19). Evobrutinib is a novel, highly selective, and covalent BTK inhibitor (20), with promising results in a positive phase II clinical trial for relapsing MS (21) and suppressive effects on B cell maturation and antigen-presenting function in experimental autoimmune encephalomyelitis (22). How BTK activity corresponds to the differentiation of CXCR3-expressing (T-bet^+^) B cells in MS and whether evobrutinib targets the brain-homing and antibody-producing potential of these B cells is unknown.

In this study, we compared BTK expression and phosphorylation in blood B cell subsets from control and clinical MS groups; these groups included clinically isolated syndrome (CIS), relapsing-remitting MS (RRMS) before and after treatment with natalizumab (NTZ; anti–VLA-4 antibody), secondary progressive MS (SPMS), and primary progressive MS (PPMS). In human B cells, BTK phosphorylation was triggered by anti-IgM, CD40L, IL-21, IFN-γ, and TLR9 ligand CpG-ODN and correlated with T-bet and CXCR3 expression levels. Class switching, brain endothelial transmigration, and memory recall responses were addressed in the context of T-bet^+^ expressing B cells with and without the use of evobrutinib in vitro.

## Results

### BTK activity is increased and less inducible in blood B cells from distinct MS subgroups.

**Figure 1:**
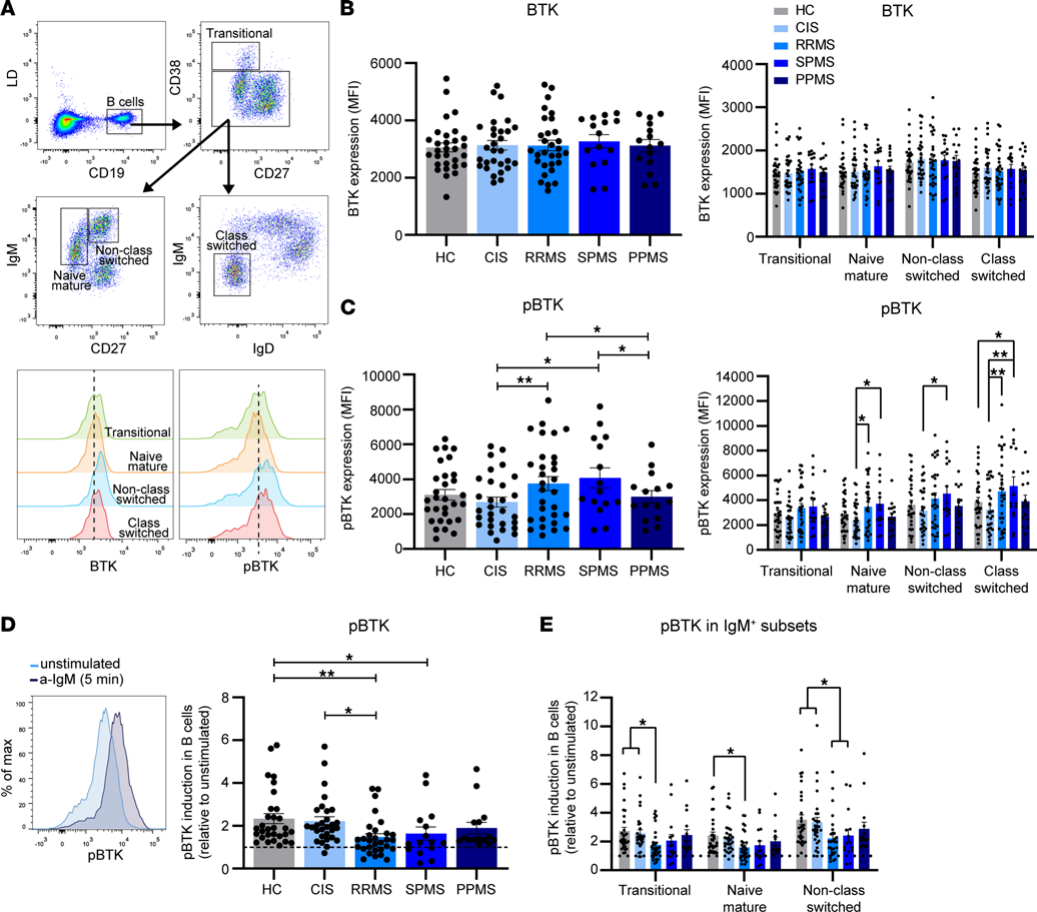
Phospho-BTK is upregulated and less induced in B cells from patients with RRMS and SPMS. (**A**) FACS gating strategy used to analyze total BTK protein and phospho-BTK (pBTK) expression levels (MFI) in transitional (CD38^hi^CD27^–^), naive mature (CD38^dim/–^IgM^+^CD27^–^), non–class-switched (CD38^dim/–^IgM^+^CD27^+^) and class-switched (CD38^dim/–^IgM^–^IgD^–^) B cell subsets. (**B**) Total BTK protein and (**C**) phospho-BTK levels were studied in blood B cells (left, total; right, subsets) from healthy controls (HCs; *n* = 30) and different MS patient groups, including clinically isolated syndrome (CIS; *n* = 29), relapsing-remitting MS (RRMS; *n* = 30), secondary progressive MS (SPMS; *n* = 15), and primary progressive MS (PPMS; *n* = 15). The inducibility of phospho-BTK in (**D**) total B cells and (**E**) IgM^+^ subsets was compared between patient and control groups using anti-IgM for 5 minutes. These FACS data were collected in 3 independent experiments, with 5–10 samples from controls and each patient group per experiment. Data are presented as the mean ± SEM. Two-way ANOVA with Fisher’s least significant difference post hoc test was performed. *P < 0.05, **P < 0.01.

To address whether BTK is differentially regulated in B cells during the pathogenesis of MS, we compared BTK protein expression and phosphorylation in ex vivo peripheral blood B cells from the healthy control (HC), CIS, RRMS, SPMS, and PPMS subgroups (Figure 1, A–C, and Supplemental Table 1; supplemental material available online with this article; https://doi.org/10.1172/jci.insight.160909DS1). Transitional (IgM^+^CD27^–^CD38^hi^), naive mature (IgM^+^CD27^–^CD38^–/dim^), and both non–class-switched (IgM^+^CD27^+^) and class-switched (IgM^–^IgD^–^) subsets were analyzed separately (Figure 1A). In contrast to comparable levels of BTK protein (Figure 1B), levels of phosphorylated BTK (phospho-BTK) were significantly higher in the RRMS and SPMS groups compared with class-switched B cells from the CIS and HC groups (Figure 1C). For the RRMS group, this was primarily observed for class-switched subsets, whereas this was the case for all subsets except transitional B cells in the SPMS group. BTK protein and phospho-BTK levels did not correlate with age (Supplemental Figure 1) or clinical parameters, such as disease duration, time to and between the first and second attack, MRI activity, and time to conversion to secondary progression (data not shown) in these MS subgroups. Ratios of phospho-BTK and BTK protein expression showed similar, but less significant differences (Supplemental Figure 2). We next compared the inducibility of phospho-BTK in B cells by stimulating them with anti-IgM F(ab′)_2_ fragments for 5 minutes. Although phospho-BTK was induced in all our groups, phospho-BTK was significantly reduced in the RRMS and SPMS groups (Figure 1D). This attenuated inducibility was similar for all IgM^+^ subsets (transitional, naive mature, and non–class-switched memory B cells; Figure 1E). These differences in anti-IgM–induced phospho-BTK were not explained by changes in IgM expression prior to stimulation (Supplemental Figure 3). These data reveal that phospho-BTK levels and inducibility are altered in B cells from patients with the relapsing and secondary progressive form of MS.

### BTK protein and phosphorylation levels correspond to CXCR3 expression in B cells from patients with MS.

**Figure 2:**
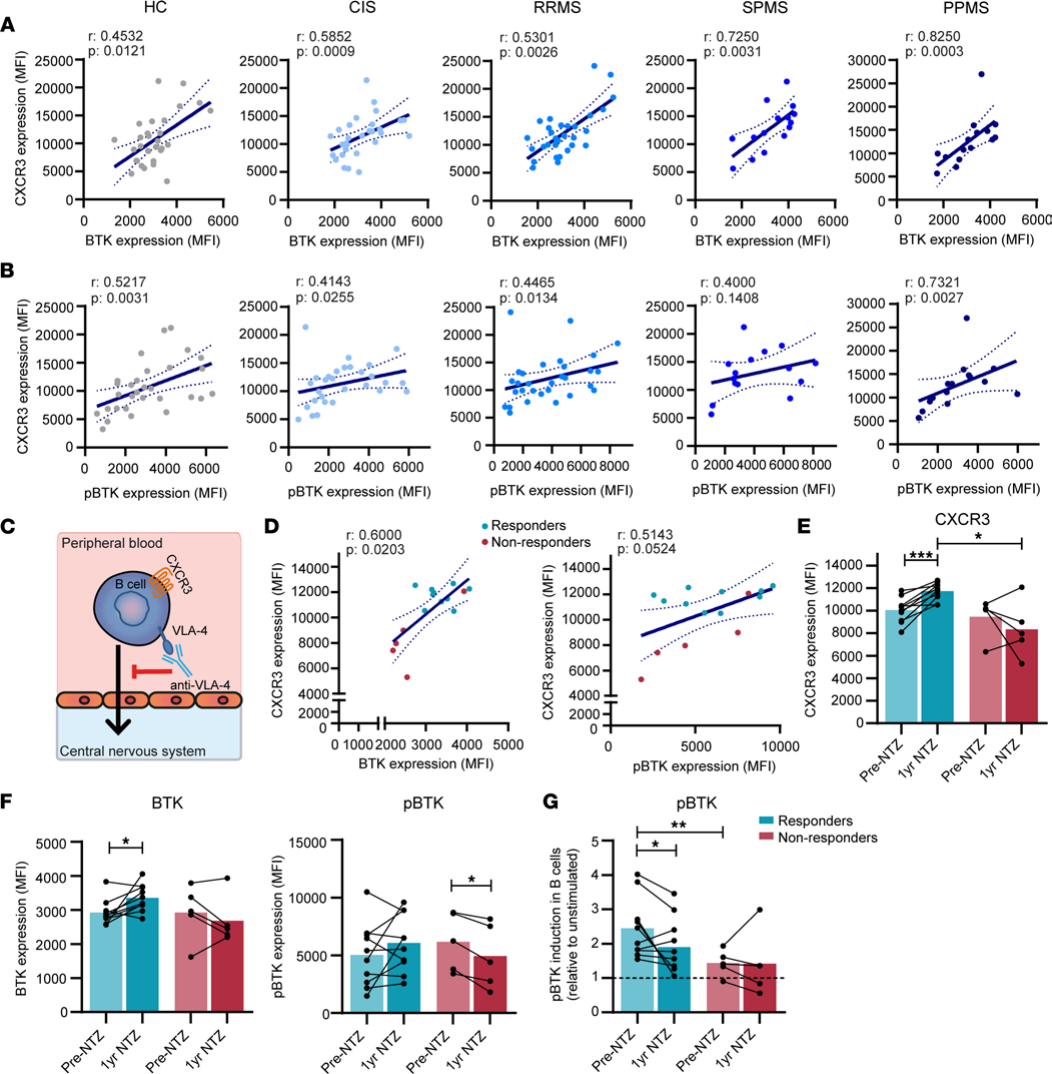
Correlation of BTK and phospho-BTK with CXCR3 expression levels in B cells from patients with MS. The association of (**A**) total BTK protein and (**B**) phospho-BTK with CXCR3 expression was analyzed for blood B cells from healthy control (HC; *n* = 30), clinically isolated syndrome (CIS; *n* = 29), relapsing-remitting MS (RRMS; *n* = 30), secondary progressive MS (SPMS; *n* = 15), and primary progressive MS (PPMS; *n* = 15) groups. FACS data were collected in the same number of experiments as depicted in Figure 1. (**C**) Graphical illustration of natalizumab (NTZ; anti–VLA-4 monoclonal antibody) treatment blocking the migration of CXCR3^+^ B cells into the CNS. (**D**–**F**) Total BTK protein, phospho-BTK, and CXCR3 expression levels were assessed in ex vivo blood B cells from NTZ-treated patients with MS. These were compared between samples from before and 1 year after treatment as well as clinical responders (*n* = 10) and nonresponders (*n* = 5). (**G**) The inducibility of phospho-BTK in B cells from responders and nonresponders before and after NTZ treatment. For the NTZ-treated MS cohort, FACS data were collected in 1 experiment with all patients included. (**A**, **B**, and **D**) Spearman’s correlations and (**E**–**G**) both paired *t* test and Mann-Whitney *U* tests were performed. *P < 0.05, **P < 0.01, ***P < 0.001.

To explore if BTK plays a role in the differentiation of B cells that are poised to infiltrate the MS brain (8), we first determined its association with CXCR3 expression in ex vivo B cells from the patient groups. Both BTK protein and phospho-BTK positively correlated with CXCR3 levels; this was seen across all patient groups and included HCs (Figure 2, A and B) and, thus, likely represents a generic feature of B cells. Overall, weaker but similar trends were found for VLA-4 but not for CXCR4 and CXCR5 (Supplemental Figure 4A). Given that NTZ (anti–VLA-4 antibody) is an effective MS drug, which causes peripheral accumulation of possibly brain-homing CXCR3^+^ B cells (Figure 2C) (8), we next studied how BTK protein levels and phosphorylation coincided with CXCR3 expression in B cells from NTZ-treated patients with MS. We found a positive correlation of BTK protein and phospho-BTK with CXCR3 levels in blood samples 1 year after treatment (Figure 2D). This was not seen for CXCR4 and CXCR5 (Supplemental Figure 4B). CXCR3 expression levels were higher in B cells from clinical responders after treatment compared with nonresponders (Figure 2E). In addition, in only clinical responders, CXCR3 was upregulated in B cells after treatment compared with B cells before treatment (Figure 2E), which further underlined our previous findings (8). This was also the case for BTK protein and, to a lesser extent, phospho-BTK (Figure 2F). Finally, phospho-BTK was less inducible by anti-IgM stimulation in B cells from nonresponders (Figure 2G), a finding that supports our earlier observations (Figure 1D). These results imply that BTK acts as a regulator of CXCR3^+^ B cell differentiation.

### BTK activity contributes to T-bet expression, and in vitro use of evobrutinib attenuates class switching in human B cells.

**Figure 3:**
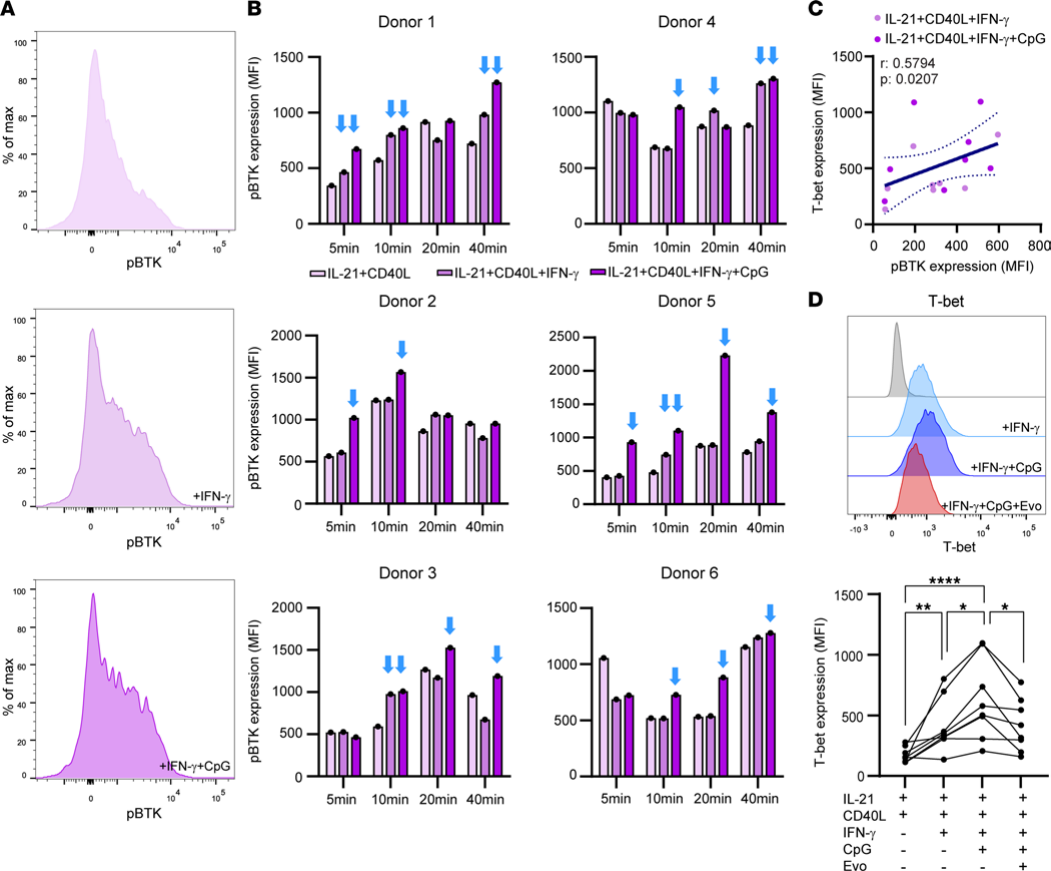
BTK activity is associated with IFN-γ– and CpG-mediated T-bet induction in human B cells. (**A**) Representative histograms of phospho-BTK expression in healthy blood-derived B cells stimulated with IL-21^+^CD40L, IL-21^+^CD40L^+^IFN-γ, and IL-21^+^CD40L^+^IFN-γ^+^CpG for 10 minutes. (**B**) Donor-specific phospho-BTK induction in blood B cells under the same conditions for 5, 10, 20, and 40 minutes (*n* = 6). The blue arrows indicate time points at which phospho-BTK is upregulated by IFN-γ with and without CpG. These FACS data were collected in 4 independent experiments, with 1–2 donors per experiment. (**C**) Correlation between phospho-BTK and T-bet levels in B cells under IL-21/CD40L/IFN-γ–inducing conditions with and without CpG for 48 hours. (**D**) The effect of evobrutinib (Evo) on IFN-γ– and CpG-induced T-bet expression in B cells (48 hours). These data were collected in 3 independent experiments, with 2 –3 donors per experiment. (**C**) Spearman’s correlation and (**D**) repeated measures 1-way ANOVA with Fisher’s least significant difference post hoc test was performed. *P < 0.05, **P < 0.01, ****P < 0.0001.

We have previously found that the IFN-γ and TLR9 signaling pathways synergize to trigger human T-bet^+^ class-switched B cells under in vitro T follicular helper–like (Tfh-like) conditions, i.e., in the presence of CD40L and IL-21 signals (8). To determine whether BTK is actively involved in this process, we analyzed phospho-BTK in blood B cells from 6 healthy donors after stimulation with IL-21, soluble CD40L, IFN-γ, and/or CpG-ODN for 5, 10, 20, and 40 minutes. Although anti-IgM was a more robust trigger of BTK activity (Supplemental Figure 5, A and B), IFN-γ alone and/or IFN-γ together with CpG-ODN induced phospho-BTK under IL-21/CD40L–containing conditions at most time points tested, albeit with differences between individual donors (Figure 3A and B). Additionally, we found a positive correlation between phospho-BTK and T-bet levels in B cells after 48-hour stimulations with IFN-γ and IFN-γ with CpG-ODN (Figure 3C), which was also influenced by the additional presence of anti-IgM F(ab′)_2_ fragments (Supplemental Figure 5C). Both IFN-γ– and CpG-ODN–mediated upregulation of T-bet in B cells was attenuated by the BTK inhibitor evobrutinib (20) (Figure 3D), pointing to direct or indirect involvement of BTK activity in T-bet induction.

**Figure 4:**
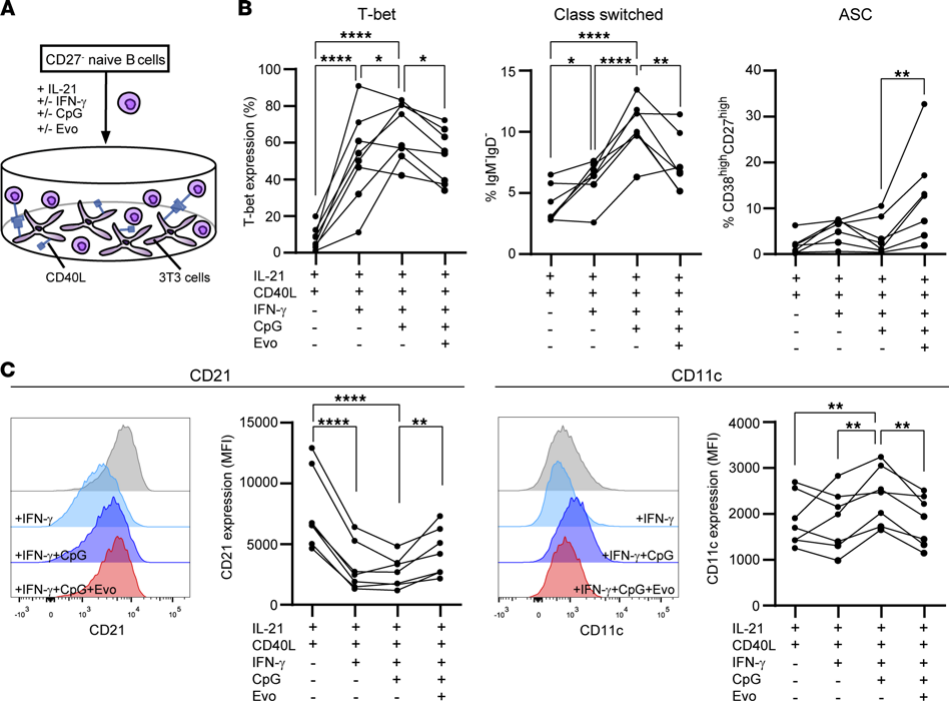
Evobrutinib interferes with the differentiation of T-bet^+^ class-switched memory subsets in naive B cell cultures. (**A**) CD27^–^ naive B cells were purified from healthy donor blood and cultured under Tfh-like conditions using IL-21 and CD40L-3T3 cells for 11 days (*n* = 7–8). IFN-γ and CpG were added with and without evobrutinib (Evo). (**B**) Proportions of in vitro–induced T-bet^+^ B cells, class-switched (IgM^–^IgD^–^) memory B cells, and antibody-secreting cells (CD38^hi^CD27^hi^; ASCs). (**C**) Histogram overlays and quantification of CD21 and CD11c expression (MFI) by these cultured B cells. Cultures were performed in 8 independent experiments, with 1 donor per experiment. Repeated measures 1-way ANOVA with Fisher’s least significant difference post hoc test were used. *P < 0.05, **P < 0.01, ****P < 0.0001.

To study further the effect of BTK on in vitro T-bet^+^ B cell differentiation, we cultured naive mature B cells with IL-21, irradiated CD40L-3T3, IFN-γ, and CpG-ODN for 11 days to induce Ig class switching (8) (Figure 4A). The addition of evobrutinib to these cultures diminished T-bet induction as well as class switching, leaving the formation of ASCs (CD38^hi^CD27^hi^) intact (Figure 4B). This was accompanied by an increase in CD21 and a decline in CD11c surface levels (Figure 4C; T-bet–associated markers) (23), thereby verifying the effect of evobrutinib on T-bet^+^ B cells. These findings indicate that differentiation of naive mature B cells into T-bet^+^ class-switched memory B cells involves BTK activity and is suppressed in vitro by the use of evobrutinib.

### The in vitro brain endothelial transmigration capacity of CXCR3^+^ class-switched memory B cells is disrupted by evobrutinib.

**Figure 5:**
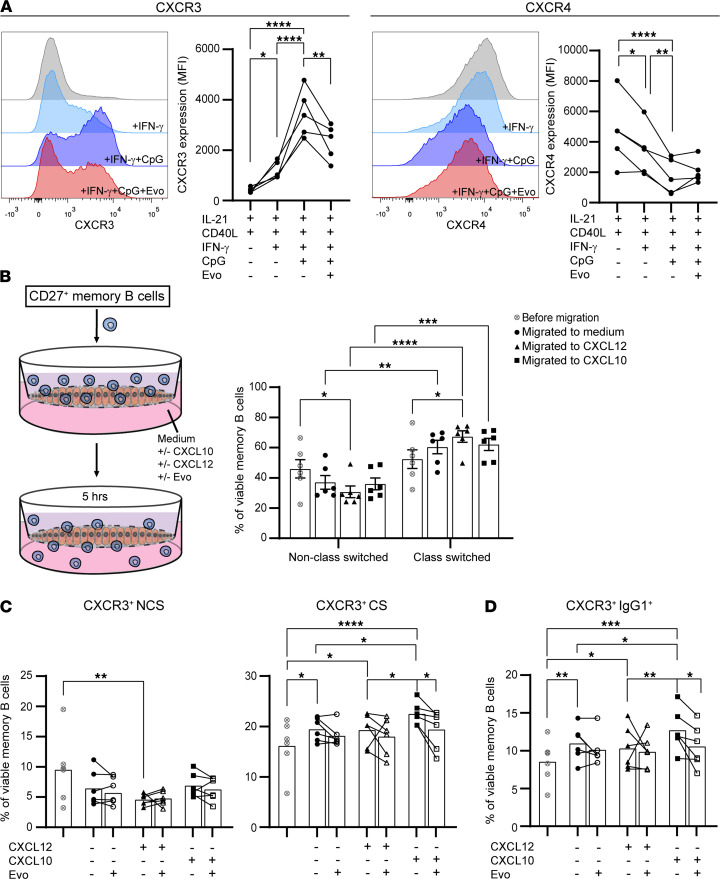
CXCL10-mediated migration of CXCR3^+^ class-switched B cells through human brain endothelial monolayers is attenuated by evobrutinib. (**A**) Histogram overlays and quantification of CXCR3 and CXCR4 expression (MFI) by B cells from 5 healthy blood donors after stimulation with IL-21, CD40L, IFN-γ, and CpG for 48 hours. Evobrutinib (Evo) was added to IFN-γ– and CpG-inducing conditions. Data were collected as described in the legend for Figure 3, C and D. (**B**–**D**) Purified CD27^+^ memory B cells from 6 healthy blood donors were assessed for their selective migration across human brain endothelial monolayers in vitro. The proportions of viable non–class-switched (IgM^+^CD27^+^; NCS), class-switched (IgM^–^CD27^+^; CS), and IgG1^+^ B cells were studied before and after migration to medium, CXCL12, and CXCL10 in the context of CXCR3 expression. These FACS data were obtained from 5 independent experiments, with 1–2 healthy donors per experiment. Data are presented as the mean ± SEM. Repeated measures 1-way ANOVA with Fisher’s least significant difference post hoc test was performed. *P < 0.05, **P < 0.01, ***P < 0.001, ****P < 0.0001.

Under Tfh-like conditions, IFN-γ– and IFN-γ/CpG-induced CXCR3 expression on B cells was significantly reduced by evobrutinib (48 hours; Figure 5A). CXCR4 and CXCR5 remained unaffected, whereas VLA-4 showed a trend toward decreased expression (Figure 5A and Supplemental Figure 6A). The evobrutinib-mediated reduction in CXCR3 seemed to be smaller in the presence of anti-IgM (Supplemental Figure 6B). This implies a selective effect of evobrutinib on CXCR3 and supports the additive effect of IFN-γ/CpG on T-bet and phospho-BTK levels without anti-IgM (Figure 3, C and D and Figure 4B). To study the potential of evobrutinib to inhibit brain-homing CXCR3^+^ memory B cells, we purified CD27^+^ memory B cells from healthy blood and analyzed subsets for their ability to cross brain endothelial monolayers in vitro (Figure 5B). Overall, class-switched B cells were better able to migrate through these monolayers than non–class-switched B cells, irrespective of CXCL10 and CXCL12 supplementation (Figure 5B). As expected, CXCR3-expressing class-switched memory (Figure 5C), and especially IgG1^+^ (Figure 5D and Supplemental Figure 6C) cells, were most attracted to CXCL10 (8). Although this effect was small, CXCL10-mediated transmigration of class-switched subsets was reduced by evobrutinib for all donors tested, an effect that was hardly encountered in the presence of CXCL12 (Figure 5, C and D). This effect of evobrutinib on the brain-homing capacity of CXCR3^+^ B cells supported the ex vivo results obtained with blood samples of NTZ-treated patients with MS (Figure 2, C–G). Although outnumbered by BTK^lo/–^ T cells, B cells expressed high levels of BTK and CXCR3 in MS CSF (Supplemental Figure 7) (8). Together, this indicates that therapeutic targeting of BTK likely halts CXCR3^+^ B cells from infiltrating the CNS in MS.

### Evobrutinib interferes with in vitro development of CXCR3^+^ memory B cell populations into ASCs.

**Figure 6:**
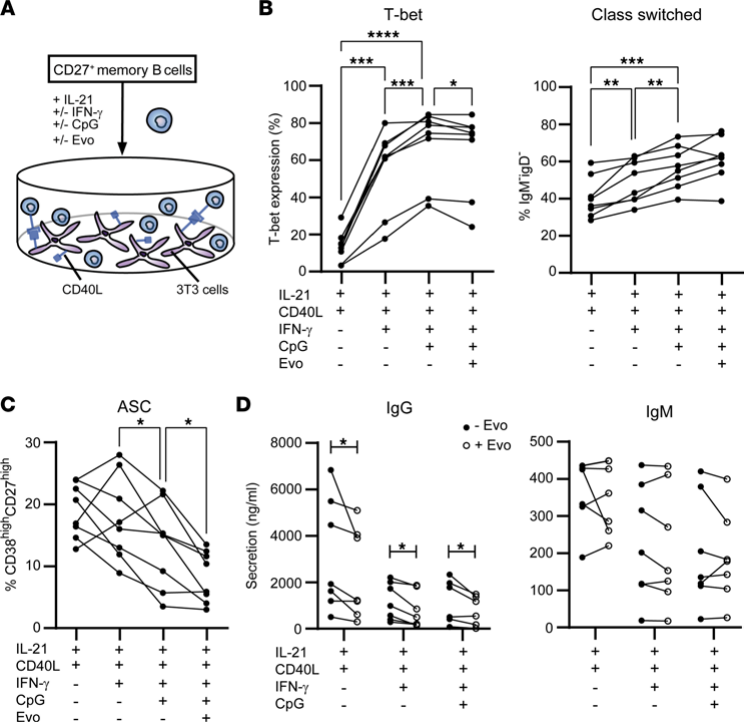
In vitro memory B cell differentiation into antibody-secreting cells is reduced by evobrutinib. (**A**) Purified CD27^+^ memory B cells from healthy blood donors were cultured under Tfh-like conditions using IL-21 and CD40L-3T3 with and without IFN-γ, CpG, and/or evobrutinib (Evo) for 6 days. The percentages of (**B**) T-bet^+^ and class-switched (IgM^–^IgD^–^) memory B cells and (**C**) antibody-secreting cells (CD38^hi^CD27^hi^; ASC) were analyzed for 8 donors. FACS data were collected as described for Figure 4. (**D**) IgG and IgM secretion within the supernatants of these cultures, as determined by ELISA (*n* =7). ELISA measurements were performed in 2 independent experiments, with 3–4 samples per experiment. (**B** and **C**) repeated measures 1-way ANOVA with Fisher’s least significant difference post hoc tests and (**D**) Wilcoxon’s signed-rank tests were used. *P < 0.05, **P < 0.01, ***P < 0.001, ****P < 0.0001.

Evobrutinib attenuated T-bet–associated class switching but not plasmablast induction in vitro using human naive B cells (Figure 4B). Nonetheless, upon reaching the MS brain, CXCR3^+^ class-switched memory B cells are likely reactivated to further develop into ASCs (13–15). To explore whether such a recall response is affected by BTK activity, we cultured CD27^+^ memory B cells under IL-21/CD40L–stimulating conditions for 6 days with and without evobrutinib (Figure 6A). Both T-bet expression and class switching were induced by IFN-γ and CpG-ODN (Figure 6B), which was also seen during naive B cell cultures. In the presence of evobrutinib, memory B cells had slightly lower T-bet levels and were less able to develop into ASCs (CD38^hi^CD27^hi^), while class switching remained unaltered (Figure 6, B and C). This suppressive effect on ASC formation was supported by a marked reduction in IgG secretion (Figure 6D). IgM secretion was not affected by evobrutinib under these circumstances (Figure 6D).

## Discussion

For optimal use of newly identified immunotherapies, it is important to uncover the exact role of biological targets in diseases such as MS. In this study, we investigated the association of BTK and its suppression by the highly selective and clinically effective drug evobrutinib with the development of pathogenic human B cells. We demonstrated that BTK activity is enhanced and less inducible in circulating B cells from patients with MS. In addition, the association of BTK with CXCR3 and T-bet upregulation as well as the reducing effects of evobrutinib in vitro indicate that BTK promotes the development of class-switched memory B cells in a T-bet–related manner. The accumulation of ex vivo BTK^+^CXCR3^+^ B cells in the blood of clinical NTZ responders as well as the impairment of in vitro CXCR3^+^ memory B cell migration across the blood-brain barrier and the maturation of these cells into ASCs by evobrutinib further highlight the potential of BTK as a therapeutic target in MS.

The reduced risk of side effects and their high potential to penetrate the CNS make next-generation BTK inhibitors promising candidates for treatment of MS (24). Such inhibitors could affect local interaction between B and T cells (22, 25), which accumulate within the perivascular space and meninges (26). Because intrathecal production of oligoclonal IgG is one of the hallmarks of MS, regardless of the pathogenicity of these antibodies, interference of BTK inhibitors with local differentiation of memory B cells into ASCs could be pathologically meaningful. The latter is especially true for patients in the advanced, progressive stages of MS, in which the disease has been postulated to be compartmentalized within the CNS, while targeting of circulating lymphocytes could be more relevant in the early phases of relapsing MS. This assumption is supported by the current selective upregulation of phospho-BTK in circulating B cells from patients with RRMS and SPMS and not patients with the PPMS. This could be relevant and should be further investigated for determining the types of patients who would benefit from treatment with BTK inhibitors. Results from ongoing clinical trials in SPMS and PPMS with various BTK inhibitors have not yet been published, but the successful phase II evobrutinib trial in MS included SPMS cases with superimposed relapses (NCT02975349) (21). Although we did not find a direct association with disease parameters, our findings support the notion that altered BTK activity influences the function of pathogenic B cells interacting with T cells as driver of MS (21, 22, 25).

Irrespective of their mode of action, the efficacy of BTK inhibitors such as evobrutinib at least partly relies on how BTK in B and myeloid cells is regulated and triggers pathogenic subsets, which may differ between autoimmune diseases. Despite enhanced BTK expression in B cells from patients with systemic lupus erythematosus (SLE) and anti-citrullinated protein antibody^+^ (ACPA^+^) but not ACPA^–^ rheumatoid arthritis (RA) (27, 28), evobrutinib only showed mild effects in SLE and RA clinical trials (NCT02975336 and NCT03233230, respectively). This is in sharp contrast to patients with MS, who had markedly fewer gadolinium-enhancing lesions after receiving evobrutinib (NCT02975349) (21). There could be several reasons for these contradicting observations. We found that phospho-BTK, but not BTK protein expression, was increased and correlated with CXCR3 expression in ex vivo B cells from patients with MS. In B cells from patients with ACPA^+^ RA, both phospho-BTK (Y551) and BTK protein levels were found to be higher (27). This implies that in MS alternative mechanisms underlie BTK dysregulation in B cells as compared with primarily antibody-driven autoimmune diseases. This is supported by observations that central B cell tolerance mechanisms are defective in RA, SLE, and neuromyelitis optica spectrum disorder but not MS (29). Moreover, BCR signaling molecules upstream of BTK may be differentially controlled in B cells between diseases, e.g., PTEN and LYN in SLE (30, 31) and SYK and CBL-B in MS (32–34). The increased BTK activity across all B cell subsets from patients with MS could indicate either a cause or consequence of their escape from peripheral tolerance (3), which seems to depend more on BTK than central tolerance (35) and needs more investigation in the future. It has already been shown that evobrutinib is effective in attenuating BTK activity in BCR-induced human B cells (22). We found reduced IgM-specific stimulation of phospho-BTK in B cells from patients with MS, suggesting that maximum phosphorylation levels are reached earlier. In another report, a similar trend was observed after triggering B cells from patients with MS with both anti-IgM and -IgG (22). This implies that from the naive stage onward, B cells need fewer external signals to differentiate into T-bet^+^ memory and ASC populations in MS (8, 12).

Phospho-BTK levels were further upregulated in ex vivo class-switched memory B cells, which is in line with previous studies (22, 27). Before memory differentiation occurs, naive B cells take up, process, and present antigen via the BCR to interact with Tfh cells at follicular borders within secondary lymphoid organs. B cell differentiation is further guided by not only costimulatory molecules, such as CD40L, but also third signals, such as Tfh cell–derived IL-21. In this phase, B cell–intrinsic T-bet induction seems to be mediated by concomitant IL-21, IFN-γ, and TLR9 signaling pathways (36–38). Our results indicate that IFN-γ and TLR9 signals, at least partly, depend on BTK activity for upregulation of T-bet, thereby triggering CXCR3 expression and Ig class switching in vitro. Such a role of BTK was also found for autoreactive B cells in SLE mice (39). Although the underlying molecular mechanism remains to be defined, proto-oncogene ETS-1 may be one of the determining factors. ETS-1 is downregulated after TLR9 ligation, a process that is controlled by BTK (40). Because ETS-1 plays a role in T-bet induction (41, 42), IFN-γ stimulation possibly overcomes TLR9-mediated reduction in ETS-1 so that it can act together with T-bet to promote Ig class switching. The synergy between IFN-γ and TLR9 responses in B cells has been proposed to regulate peripheral tolerance (43), which is disturbed in MS (3). The observed inhibitory effects of evobrutinib on IFN-γ– and CpG-ODN–mediated class switching in B cells is relevant, given their enhanced responsiveness to such signals and capacity to serve as potent antigen-presenting cells in MS (4, 8, 12). This is supported by the fact that the antigen-presenting cell function of B cells is reinforced by T-bet (8, 44) as well as BTK (45, 46) and is impaired in experimental autoimmune encephalomyelitis mice following evobrutinib treatment (22).

One limitation of our in vitro experiments is that we did not take into account a possible survival advantage of T-bet^+^ class-switched B cells and ASCs. The observed in vitro effects of evobrutinib also need to be confirmed in vivo. The suppressive effect of evobrutinib on class-switched B cells crossing blood-CNS barriers in vitro is at least in line with the role of BTK in B cell tissue homing (47). BTK is known to mediate CXCR4 and CXCR5 signaling (48), but our current finding that it can control the CXCR3/CXCL10 axis is possibly new. With CXCL10, CXCL12, and CXCL13 being locally enriched, CXCR4 and CXCR5 can be expected to be more involved in the organization, while CXCR3 may be more related to the recruitment of B cells in the MS brain (8). The increased BTK activity in CXCR3^+^ class-switched memory B cells from patients with MS puts this pathogenic subset forward as a candidate target of evobrutinib to prevent B cell recruitment in the CNS. This increase was found in B cells from both patients with RRMS and SPMS but not patients with PPMS, pointing to a direct relation with local inflammation rather than neurodegeneration. CNS-infiltrating CXCR3^+^ memory B cells are also poised to mature into ASCs in vitro (13, 14), which seems to be a process that can be affected by evobrutinib (this study). Hence, the potential of next-generation BTK inhibitors to penetrate the CNS may provide an opportunity to not only prevent, but also target (clinically relevant) local antibody production in patients with MS (15), which should be investigated in the near future. In addition, such inhibitors may be feasible as treatment options in patients who have to discontinue NTZ treatment. By exposing the disease-relevant mechanisms involved, this work reveals that BTK inhibition is a therapeutic approach that meets the need for selectively targeting pathogenic B cells in MS and other types inflammatory and autoimmune disorders (17, 21, 49).

## Methods

### Patients and sampling.

In a first screen, we analyzed PBMCs from 29 patients with CIS, 30 patients with RRMS, 15 patients with SPMS, and 15 patients with PPMS as well as 30 HCs. None of these patients received any disease-modifying treatment or methyl prednisolone for at least 3 months before sampling. In a second screen, we included 15 patients with RRMS who were treated with NTZ for 12 months (responders, *n* = 10; nonresponders, *n* = 5). Responders to NTZ were defined as treated patients who remained free of clinical attacks, i.e., subacute worsening of existing symptoms, or new symptoms after at least 30 days of improvement or stable disease (50). For 1 patient with MS, we were able to analyze B cells in both CSF and blood. Patients and controls were included at the MS Center ErasMS. Clinical characteristics are shown in Supplemental Table 1. CIS was defined as a first clinical attack of demyelination in the CNS. All patients with CIS were sampled within 4 months after their first attack. Patients with MS were diagnosed according to the McDonald 2017 criteria (51). For our functional studies, buffy coats (Sanquin) were obtained from healthy volunteers.

Peripheral blood was collected using Cell Preparation Tubes (BD Biosciences) containing sodium heparin. PBMCs were isolated according to the manufacturer’s instructions. PBMCs were taken up in RPMI 1640 (Lonza) containing 20% FCS (Thermo Fisher Scientific) and 10% DMSO (MilliporeSigma) and stored in liquid nitrogen until further use.

### Antibodies and flow cytometry.

The details of anti-human monoclonal antibodies used for flow cytometric analysis of B cells are included in Supplemental Table 2. For our first ex vivo screen, we measured B cells from HCs and patients with CIS, RRMS, SPMS, and PPMS within the same experiment, divided equally over 3 experiments. B cells from NTZ-treated patients with RRMS were measured in a separate experiment. For the induction of phospho-BTK (Y223) in B cells, PBMCs were treated for 5 minutes with F(ab′)_2_ anti-human IgM (20 μg/ml; Southern Biotech). For in vitro differentiation, total (CD19^+^CD3^–^), naive (CD19^+^CD3^–^CD27^–^), and memory (CD19^+^CD3^–^CD27^+^) B cells were purified using a FACSAria III sorting machine (BD Biosciences). For extracellular staining, cells were incubated with antibody mix for 30 minutes at 4°C. For intracellular staining of phospho-BTK and T-bet, cells were fixed and permeabilized using the eBioscience FoxP3/Transcription Factor Staining Buffer Kit (Thermo Fisher Scientific) and incubated with an antibody mix for 30 minutes at 4°C. For intracellular BTK staining, cells were first incubated for extracellular marker staining as described above. After extracellular staining, cells were fixed with 2% PFA for 10 minutes at 4°C, permeabilized in 0.5% saponine buffer for 10 minutes at 4°C, followed by intracellular staining in 0.5% saponine buffer for 60 minutes at 4°C. After staining, cells were suspended and measured on an LSRFortessa flow cytometer (BD Biosciences). Data were analyzed using FACSDiva 8.1 and FlowJo v10 software (both BD Biosciences). Dependent on the distribution per marker, we used mean (nonskewed) or median (skewed) to indicate fluorescence intensity (MFI).

### Short-term B cell stimulation.

For the 5- to 40-minute in vitro stimulation experiments, the following B cell stimuli were used: F(ab′)_2_ goat anti-human IgM (20 μg/ml; Southern Biotech), rhIL-21 (50 ng/ml; Thermo Fisher Scientific), soluble CD40L (0.1 μg/ml; Enzo Life Sciences), rhIFN-γ (50 ng/ml; Peprotech), and CpG-ODN (10 μg/ml; Invivogen). For these stimulations, we used and adapted a protocol described in a recent study (52). In short, 5 × 10^5^ PBMCs were thawed and put on ice in RPMI containing 2% FCS using 96-well round-bottom plates. For 5- and 10-minute stimulations, live/dead viability stain was added together with the stimuli. For 20- and 40-minute stimulations, cells were first incubated with a live/dead viability stain for 15 minutes at 4°C, washed, and then suspended with stimuli. After stimulation at 37°C, cells were placed on ice, suspended in cold eBioscience Fix/Perm buffer (Thermo Fisher Scientific), and fixed for 15 minutes (4°C) prior to the staining according to above described protocols. For the 48-hour stimulations, the following B cell stimuli were used: F(ab′)_2_ goat anti-human IgM (10 μg/ml; Jackson ImmunoResearch), rhIL-21 (50 ng/ml; Thermo Fisher Scientific), soluble CD40L (0.1 μg/ml; Enzo Life Sciences), rhIFN-γ (50 ng/ml; Peprotech), and CpG-ODN (10 μg/ml; Invivogen). 5 × 10^5^ B cells were suspended in RPMI containing 5% FCS, β-mercaptoethanol (MilliporeSigma), L-glutamin (Lonza), and apo-transferrin (MilliporeSigma); they were treated with and without evobrutinib (1 μM; EMD Serono, Billerica) in 48-well plates.

### Long-term B cell cultures.

In vitro naive and memory B cell differentiation assays were performed as reported previously (8, 13). In short, irradiated 3T3 fibroblasts expressing human CD40L were cocultured with purified naive mature (CD19^+^CD38^–/dim^CD27^–^; primary response) or memory (CD19^+^CD38^–/dim^CD27^+^; recall response) B cells in the presence of rhIL-21 (50 ng/ml; Thermo Fisher Scientific). For naive B cell cultures, rhIFN-γ (50 ng/ml; Peprotech) was added with and without CpG-ODN (10 μg/ml; Invivogen) to induce class switching and ASC development, respectively (8). After 6 (recall response) or 11 (primary response) days of culturing, viable (live/dead^–^) CD19^+^ cells were examined using flow cytometry. The supernatants were collected and stored at –80°C until further use.

### IgM and IgG ELISA.

Flat-bottom 96-well plates (Corning) were coated overnight with goat anti-human Ig (1 mg/ml; Southern Biotech) at 4°C, washed with PBS/0.05%Tween-20, and blocked with PBS/5% FCS for 2 hours at room temperature. Samples were added for 1.5 hours. After washing, peroxidase-conjugated goat anti-human IgG (Thermo Fisher Scientific) or rabbit anti-human IgM (Jackson ImmunoResearch) was used to detect bound antibody. TMB Substrate (Thermo Fisher Scientific) was used to reveal peroxidase activity. Reactions were stopped with sulfuric acid and optical densities were measured at 450 nm using a BioTek Synergy 2 reader (Winooski). Concentrations were calculated using standard curves that were generated for each assay.

### B cell transmigration assay.

For transmigration experiments, 2.5 × 10^5^ to 5 × 10^5^ CD27^+^ memory B cells isolated from buffy coats were placed on confluent monolayers of human brain endothelial cells (hCMEC/D3) (53) on 5 μm pore size Transwell plates (Corning Life Sciences) coated with collagen. B cell migration toward medium, CXCL10 (900 ng/ml; R&D Systems; ref. 8), or CXCL12 (100 ng/ml; R&D Systems) was analyzed after 5 hours at 37°C. Percentages of memory B cell subsets were compared before and after transmigration using flow cytometry.

### Statistics.

Statistical analyses were performed using Graphpad Prism 9 and are described in each figure legend. Statistical tests included 2-way ANOVA with Fisher’s least significant difference post hoc test, repeated measures 1-way ANOVA with Fisher’s least significant difference post hoc test with Spearman’s correlation, Spearman’s correlation, paired 2-tailed *t* test, Mann-Whitney *U* test, and Wilcoxon’s signed-rank tests. Both percentages and MFI are depicted as individual data points together with the corresponding mean. *P* values of less than 0.05 were considered statistically significant.

### Study approval.

All patients and controls gave written informed consent, and study protocols were approved by the medical ethics committee of Erasmus MC.

## Author contributions

LR performed experiments, analyzed data, interpreted results, and revised the manuscript. JVL set up in vitro B cell differentiation and transmigration assays, interpreted results, generated displayed items, and revised the manuscript. Regarding the shared contribution, the authorship order for LR and JVL has been determined by time of involvement in the research: LR initiated the study and JVL finished the study. LB and MJM performed experiments. LB, MJM, and SCK analyzed data. LB and SCK interpreted data. KMB coordinated clinical studies and looked up patient characteristics. AFWW performed experiments. HEDV provided material and input for the migration assay. JR, OBJC, and RWH shared optimal protocols for BTK detection and revised the manuscript. RG and UB provided the BTK inhibitor. RG, UB, and JS provided intellectual input. UB and JS critically revised the manuscript. JS coordinated the outpatient clinic, supervised the clinical studies, and obtained funding. MMVL designed the research, obtained funding, discussed results, supervised the project team, and wrote the manuscript.

## Supplementary Material

Supplemental data

## Version 1 07/19/2022

In-Press Preview

## Version 2 08/22/2022

Electronic publication

## References

[1] Sabatino JJ (2019). B cells in autoimmune and neurodegenerative central nervous system diseases. Nat Rev Neurosci.

[2] Cencioni MT (2021). B cells in multiple sclerosis - from targeted depletion to immune reconstitution therapies. Nat Rev Neurol.

[3] Kinnunen T (2013). Specific peripheral B cell tolerance defects in patients with multiple sclerosis. J Clin Invest.

[4] Jelcic I (2018). Memory B cells activate brain-homing, autoreactive CD4^+^ T cells in multiple sclerosis. Cell.

[5] Bjornevik K (2022). Longitudinal analysis reveals high prevalence of Epstein-Barr virus associated with multiple sclerosis. Science.

[6] Lunemann JD (2008). EBNA1-specific T cells from patients with multiple sclerosis cross react with myelin antigens and co-produce IFN-gamma and IL-2. J Exp Med.

[7] Wang J (2020). HLA-DR15 molecules jointly shape an autoreactive T cell repertoire in multiple sclerosis. Cell.

[8] van Langelaar J (2019). Induction of brain-infiltrating T-bet-expressing B cells in multiple sclerosis. Ann Neurol.

[9] Piovesan D (2017). c-Myb Regulates the T-bet-dependent differentiation program in B cells to coordinate antibody responses. Cell Rep.

[10] Rawlings DJ (2017). Altered B cell signalling in autoimmunity. Nat Rev Immunol.

[11] Rivera-Correa J (2017). Plasmodium DNA-mediated TLR9 activation of T-bet^+^ B cells contributes to autoimmune anaemia during malaria. Nat Commun.

[12] Bar-Or A (2010). Abnormal B-cell cytokine responses a trigger of T-cell-mediated disease in MS?. Ann Neurol.

[13] van Langelaar J (2021). The association of Epstein-Barr virus infection with CXCR3(+) B-cell development in multiple sclerosis: impact of immunotherapies. Eur J Immunol.

[14] Janssen M (2021). Pregnancy-induced effects on memory B-cell development in multiple sclerosis. Sci Rep.

[15] Fransen NL (2021). Absence of B cells in brainstem and white matter lesions associates with less severe disease and absence of oligoclonal bands in MS. Neurol Neuroimmunol Neuroinflamm.

[16] Timofeeva N, Gandhi V (2021). Ibrutinib combinations in CLL therapy: scientific rationale and clinical results. Blood Cancer J.

[17] Neys SFH (2021). Targeting Bruton’s tyrosine kinase in inflammatory and autoimmune pathologies. Front Cell Dev Biol.

[18] Crofford LJ (2016). The role of Bruton’s tyrosine kinase in autoimmunity and implications for therapy. Expert Rev Clin Immunol.

[19] Rip J (2019). Toll-like receptor signaling drives Btk-mediated autoimmune disease. Front Immunol.

[20] Haselmayer P (2019). Efficacy and pharmacodynamic modeling of the BTK inhibitor evobrutinib in autoimmune disease models. J Immunol.

[21] Montalban X (2019). Placebo-controlled trial of an oral BTK inhibitor in multiple sclerosis. N Engl J Med.

[22] Torke S (2020). Inhibition of Bruton’s tyrosine kinase interferes with pathogenic B-cell development in inflammatory CNS demyelinating disease. Acta Neuropathol.

[23] Karnell JL (2017). Role of CD11c^+^ T-bet^+^ B cells in human health and disease. Cell Immunol.

[24] Contentti EC, Correale J (2020). Bruton’s tyrosine kinase inhibitors: a promising emerging treatment option for multiple sclerosis. Expert Opin Emerg Drugs.

[25] Bhargava P (2021). Imaging meningeal inflammation in CNS autoimmunity identifies a therapeutic role for BTK inhibition. Brain.

[26] Jain RW, Yong VW B cells in central nervous system disease: diversity, locations and pathophysiology. Nat Rev Immunol.

[27] Corneth OBJ (2017). Enhanced Bruton’s tyrosine kinase activity in peripheral blood B lymphocytes from patients with autoimmune disease. Arthritis Rheumatol.

[28] Kong W (2018). Increased expression of Bruton’s tyrosine kinase in peripheral blood is associated with lupus nephritis. Clin Rheumatol.

[29] Cotzomi E (2019). Early B cell tolerance defects in neuromyelitis optica favour anti-AQP4 autoantibody production. Brain.

[30] Brodie EJ (2018). Lyn, Lupus, and (B) lymphocytes, a lesson on the critical balance of kinase signaling in immunity. Front Immunol.

[31] Wu XN (2014). Defective PTEN regulation contributes to B cell hyperresponsiveness in systemic lupus erythematosus. Sci Transl Med.

[32] Sohn HW (2003). Cbl-b negatively regulates B cell antigen receptor signaling in mature B cells through ubiquitination of the tyrosine kinase Syk. J Exp Med.

[33] van Langelaar J (2020). B and T cells driving multiple sclerosis: identity, mechanisms and potential triggers. Front Immunol.

[34] Yasuda T (2002). Cbl-b positively regulates Btk-mediated activation of phospholipase C-gamma2 in B cells. J Exp Med.

[35] Satterthwaite AB (2017). Bruton’s tyrosine kinase, a component of B cell signaling pathways, has multiple roles in the pathogenesis of lupus. Front Immunol.

[36] Knox JJ (2019). T-bet^+^ memory B cells: Generation, function, and fate. Immunol Rev.

[37] Naradikian MS (2016). Cutting edge: IL-4, IL-21, and IFN-γ interact to govern T-bet and CD11c expression in TLR-activated B cells. J Immunol.

[38] Xu X (2018). Phosphorylation-mediated IFN-gammaR2 membrane translocation is required to activate macrophage innate response. Cell.

[39] Halcomb KE (2008). Btk regulates localization, in vivo activation, and class switching of anti-DNA B cells. Mol Immunol.

[40] Luo W (2014). A balance between B cell receptor and inhibitory receptor signaling controls plasma cell differentiation by maintaining optimal Ets1 levels. J Immunol.

[41] Grenningloh R (2005). Ets-1, a functional cofactor of T-bet, is essential for Th1 inflammatory responses. J Exp Med.

[42] Nguyen HV (2012). The Ets-1 transcription factor is required for Stat1-mediated T-bet expression and IgG2a class switching in mouse B cells. Blood.

[43] Sindhava VJ (2017). A TLR9-dependent checkpoint governs B cell responses to DNA-containing antigens. J Clin Invest.

[44] Rubtsov AV (2015). CD11c-expressing B cells are located at the T Cell/B cell border in spleen and are potent APCs. J Immunol.

[45] Li R (2022). BTK inhibition limits B-cell-T-cell interaction through modulation of B-cell metabolism: implications for multiple sclerosis therapy. Acta Neuropathol.

[46] Sharma S (2009). Btk regulates B cell receptor-mediated antigen processing and presentation by controlling actin cytoskeleton dynamics in B cells. J Immunol.

[47] Hendriks RW (2014). Targeting Bruton’s tyrosine kinase in B cell malignancies. Nat Rev Cancer.

[48] de Gorter DJ (2007). Bruton’s tyrosine kinase and phospholipase Cgamma2 mediate chemokine-controlled B cell migration and homing. Immunity.

[49] Lehmann-Horn K (2017). Deciphering the role of B cells in multiple sclerosis-towards specific targeting of pathogenic function. Int J Mol Sci.

[50] van Langelaar J (2018). T helper 17.1 cells associate with multiple sclerosis disease activity: perspectives for early intervention. Brain.

[51] Thompson AJ (2018). Diagnosis of multiple sclerosis: 2017 revisions of the McDonald criteria. Lancet Neurol.

[52] Rip J (2020). Phosphoflow protocol for signaling studies in human and murine B cell subpopulations. J Immunol.

[53] Weksler BB (2005). Blood-brain barrier-specific properties of a human adult brain endothelial cell line. FASEB J.

