# Supplementary material for: Human T-bet+ B cell development is associated with BTK activity and suppressed by evobrutinib
Source: JCI Insight. 2022 Aug 22;7(16):e160909. doi: 10.1172/jci.insight.160909 (PMC9462504; doi:10.1172/jci.insight.160909)
Supplement: Supplemental data [file jciinsight-7-160909-s066.pdf]

Supplementary Table 1: Patient characteristics

Ex vivo	N	Age *(mean[min-max])*	Gender *(% females)*
**HC**	30	42 [20-73]	60
**CIS**	29	33 [19-50]	72.4
**RRMS**	30	43 [19-80]	70
**SPMS**	15	55 [37-68]	53.3
**PPMS**	15	55 [38-72]	60
**NTZ-MS**	15	35 [17-52]	80
NTZ - responders	10	34 [17-49]	70
NTZ - non-responders	5	37 [18-52]	100

HC, healthy control; CIS, clinically isolated syndrome; RRMS, relapsing-remitting MS; SPMS, secondary progressive MS; PPMS, primary progressive MS, NTZ-MS, natalizumab-treated MS

Supplementary Table 2: Monoclonal anti-human antibodies used for FACS.

Marker	Label	Clone	Supplier
BTK	PE	53/BTK	BD Biosciences[A]
CD3	AF700	SK7	Biolegend[B]
CD3	APC	SK7	BD Biosciences
CD11c	BV605	B-ly6	BD Biosciences
CD19	BV786	HIB19	Biolegend
CD21	BV711	B-Ly-4	BD Biosciences
CD27	BV421	M-T271	BD Biosciences
CD38	BV605, PE-Cy7 or PerCP-Cy5.5	HIT2	Biolegend
CXCR3	PE-Cy7	G025H7	Biolegend
CXCR4	APC or PE-CF594	12G5	BD Biosciences
CXCR5	PerCP-Cy5.5	RF8B2	BD Biosciences
Fixable viability stain	AF700		BD Biosciences
IgD	PE-CF594	IA6-2	BD Biosciences
IgG	APC-H7	G18-145	BD Biosciences
IgG1	PE	HP6001	Southern Biotech[C]
IgG2	AF488	HP6002	Southern Biotech
IgM	BV510	MHM-88	Biolegend
pBTK	PE	pY223	BD Biosciences
T-bet	PE-Cy7	4B10	Biolegend
VLA-4	BB515 or BV711	9F10	BD Biosciences

[A] BD Biosciences, Erembodegem, Belgium
[B] Biolegend, London, UK
[C] Southern Biotech via ITK Diagnostics, Uithoorn, The Netherlands

Supplementary Figure 1

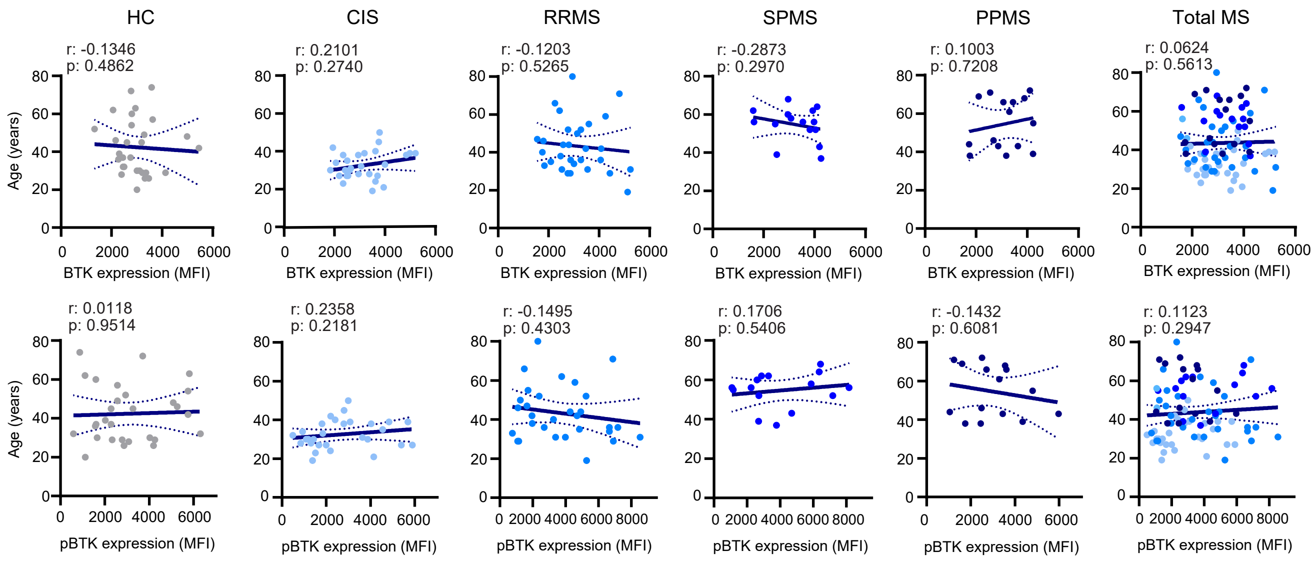

Supplementary Figure 1. No association between age and BTK and activity in B cells of MS patients and healthy individuals. Correlation of age with BTK protein and phospho-BTK levels in circulating B cells of healthy control (HC; n=30), clinically isolated (CIS; n=29), relapsing-remitting MS (RRMS; n=30) secondary progressive MS (SPMS; n=15) and primary progressive MS (PPMS; n=15) patient groups as well as all MS patients together (total MS; n=89). Data were collected from the same number of experiments as depicted in Figure 1. Spearman correlations were performed.

Supplementary Figure 2

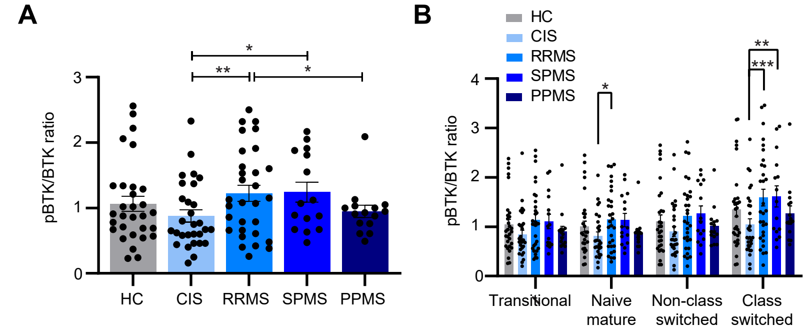

Supplementary Figure 2. pBTK/BTK ratio is increased in B cells of RRMS and SPMS patients. Phospho-BTK/BTK ratios in (**A**) total B cells as well as (**B**) transitional ($CD38^{high}CD27^{-}$), naive mature ($CD38^{dim/-}IgM^{+}CD27^{-}$), non-class switched ($CD38^{dim/-}IgM^{+}CD27^{+}$) and class-switched ($CD38^{dim/-}IgM^{-}IgD^{-}$) B-cell subsets within the blood of healthy control (HC; n=30), clinically isolated (CIS; n=29), relapsing-remitting MS (RRMS; n=30) secondary progressive MS (SPMS; n=15) and primary progressive MS (PPMS; n=15) patient groups. Data were collected from the same number of experiments as depicted in Figure 1. Data are presented as the mean ± SEM. 2-way ANOVA with Fisher's LSD post-hoc test was performed. ***p <0.001, **p <0.01 and *p <0.05.

Supplementary Figure 3

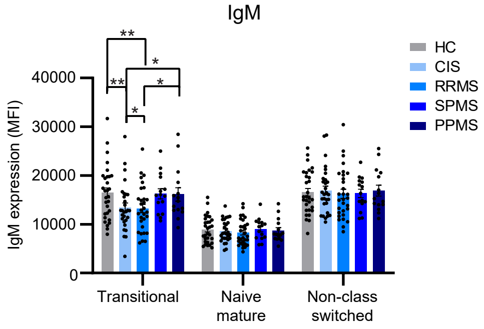

Supplementary Figure 3. IgM expression in *ex vivo* blood B-cell subsets of different MS patients and healthy controls. IgM expression levels (MFI) in transitional ($CD38^{high}CD27^{-}$), naive mature ($IgM^{+}CD27^{-}$) and non-class switched ($IgM^{+}CD27^{+}$) B-cell subsets within the blood of healthy controls (HC; n=30) and different MS patient groups, i.e. clinically isolated syndrome (CIS; n=29), relapsing-remitting MS (RRMS; n=30), secondary progressive MS (SPMS; n=15) and primary progressive MS (PPMS; n=15). Data were collected from the same number of experiments as depicted in Figure 1. Data are presented as the mean ± SEM. 2-way ANOVA with Fisher's LSD post-hoc test was performed. **p <0.01 and *p <0.05.

Supplementary Figure 4

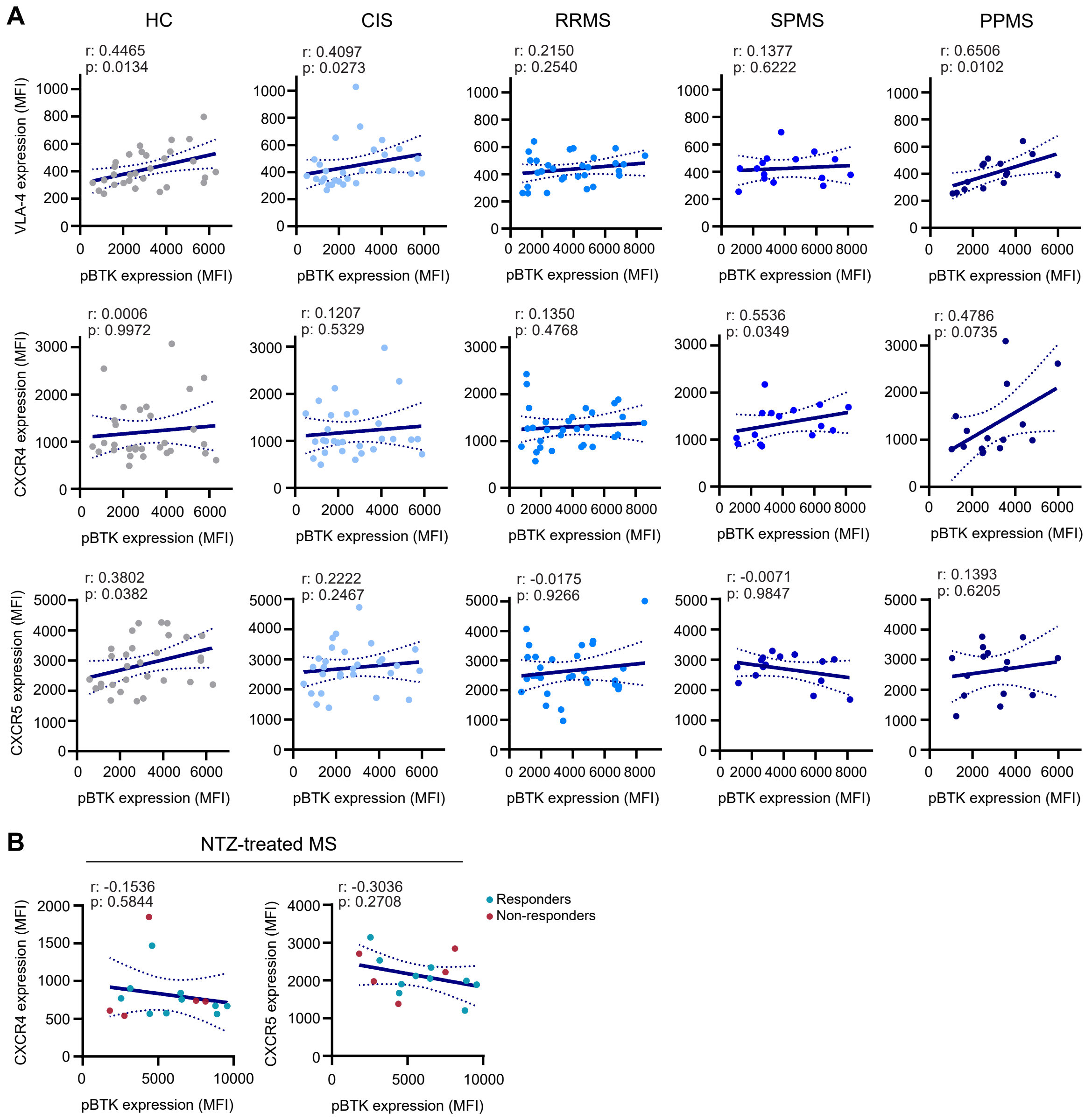

Supplementary Figure 4. The association of basal BTK activity with VLA-4, CXCR4 and CXCR5 expression in B cells of MS patients. (**A**) Correlation of BTK phosphorylation with VLA-4, CXCR4 and CXCR5 expression in circulating B cells of healthy control (HC; n=30), clinically isolated (CIS; n=29), relapsing-remitting MS (RRMS; n=30) secondary progressive MS (SPMS; n=15) and primary progressive MS (PPMS; n=15) patient groups. (**B**) Correlation of BTK phosphorylation with CXCR4 and CXCR5 expression in circulating B cells from MS patients treated with natalizumab (NTZ; n=15). Data were collected from the same number of experiments as depicted in Figures 1 and 2. Spearman correlations were performed.

Supplementary Figure 5

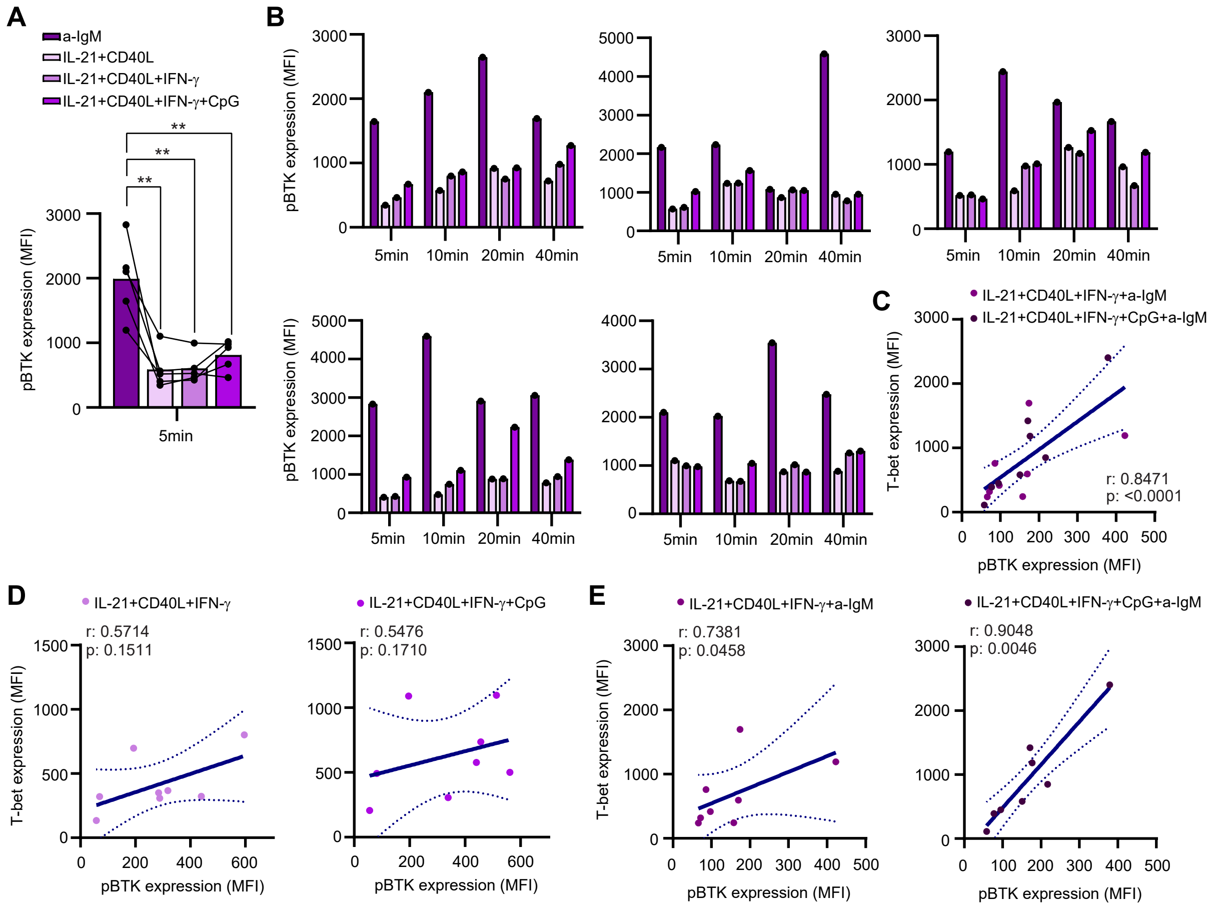

Supplementary Figure 5. The impact of anti-IgM on phospho-BTK levels during short-term stimulations. (**A**) Phospho-BTK induction in blood B cells from 5 healthy donors under anti-IgM- or IL-21/CD40L-inducing conditions with and without IFN-γ or CpG for 5 min. (**B**) Donor-specific phospho-BTK induction under the same conditions for 5, 10, 20 and 40 min. (**C**) Correlation between T-bet and phospho-BTK levels in total B cells from 8 healthy blood donors after 48 h stimulation with anti-IgM, IL-21, CD40L and IFN-γ with and without CpG. (**D-E**) Split correlations between T-bet and phospho-BTK levels in B cells under IL-21/CD40L/IFN-γ-inducing conditions with and without CpG as well as with and without anti-IgM. FACS data were collected from the same number of experiments as depicted in Figure 3. RM one-way ANOVA with Fisher's LSD post-hoc test (**A**) as well as Spearman correlations (**C-E**) were performed. **p <0.01.

Supplementary Figure 6

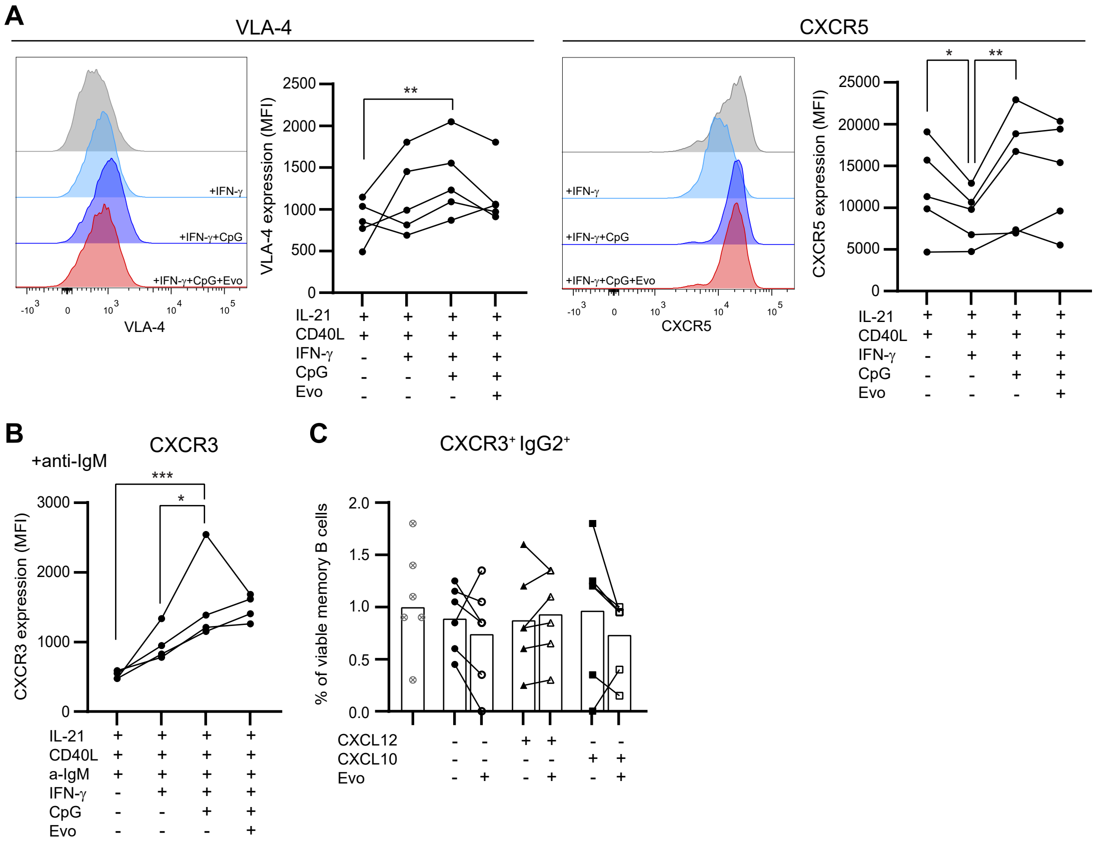

Supplementary Figure 6. ***In vitro*** **effects of evobrutinib on VLA-4 and CXCR5 expression during B-cell stimulations and the transmigration of CXCR3⁺IgG2⁺ B cells.** (**A**) Histogram overlays and quantification of the MFI of VLA-4 and CXCR5 for B cells from the blood of 5 healthy donors after 48 h stimulations with IL-21, CD40L, IFN-γ and CpG in the presence or absence of evobrutinib. (**B**) CXCR3 expression by healthy blood B cells under the same stimulating conditions with the addition of anti-IgM. (**C**) Percentages of viable CXCR3⁺IgG2⁺ B cells before and after migration to medium, CXCL12 and CXCL10 through monolayers of human brain endothelial cells (n=6). FACS data were collected from the same number of experiments as depicted in Figures 3 and 5. RM one-way ANOVA with Fisher's LSD post-hoc test was performed. ***p <0.001, **p <0.01 and *p <0.05.

Supplementary Figure 7

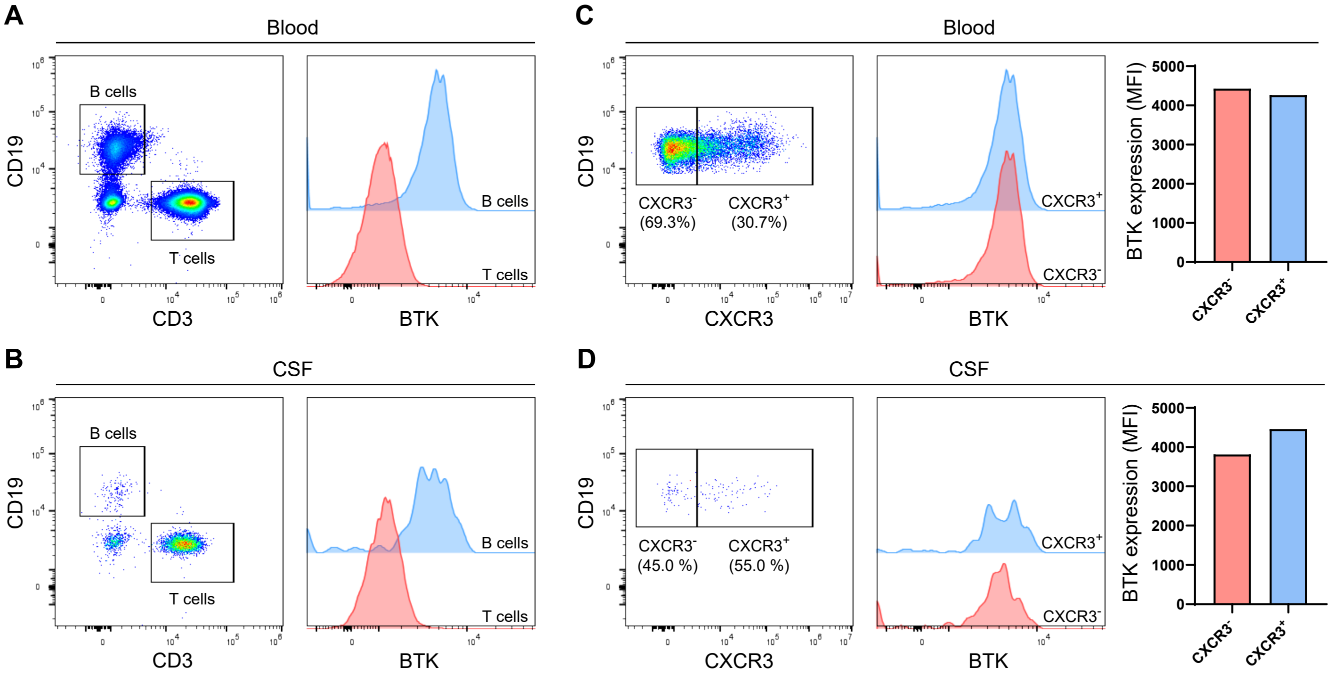

Supplementary Figure 7. BTK expression within CXCR3⁻ and CXCR3⁺ B cells from blood and CSF of an MS patient. FACS dot plots for identifying B and T cells within viable lymphocytes and histograms with total BTK expression levels within these cell populations in blood **(A)** and CSF **(B)** from an MS patient. FACS dot plots for identifying CXCR3⁻ and CXCR3⁺ B cells and histograms and bar charts with total BTK expression levels (MFI) within these cell populations in blood **(C)** and CSF **(D)** from an MS patient.
